# Regulation of E-cadherin by mixed lineage kinase 3 (MLK3) mediates cell adhesion in ovarian cancer spheroids

**DOI:** 10.1016/j.bbamcr.2026.120106

**Published:** 2026-01-07

**Authors:** Mariah C.J. Rozier, Danielle N. Adjei, Rachel A. Radtke, Deborah N. Chadee

**Affiliations:** Department of Molecular, Cellular and Developmental Biology (MCDB), University of Toledo, Toledo, OH, 43606, USA

**Keywords:** Mixed lineage kinase 3 (MLK3), Mitogen-activated protein kinase (MAPK), Ovarian cancer, E-cadherin, Cell adhesion

## Abstract

Mixed lineage kinase 3 (MLK3) is a serine/threonine mitogen-activated protein kinase (MAPK) kinase kinase (MAP3K) that activates MAPK signaling pathways and promotes cell proliferation, migration and invasion. E-cadherin is an essential cell adhesion protein that mediates cell-cell interactions in epithelial adherens junctions and is crucial for the integrity of tissues and organs. Here, we demonstrate that MLK3 siRNA knockdown or kinase inhibition significantly impaired spheroid formation in SKOV3, TOV112D and OVCAR3 ovarian cancer cells, that was rescued by overexpression of E-cadherin. Endogenous MLK3 and E-cadherin were co-immunoprecipitated from ovarian cancer cell lysates; and recombinant MLK3 and E-cadherin directly interacted *in vitro*. MLK3 phosphorylates E-cadherin and has a positive regulatory effect on E-cadherin protein stability. Like E-cadherin, MLK3 is localized to the plasma membrane at regions of cell-cell contact, and MLK3 also has cytoplasmic and nuclear localization. Neither the MLK3-E-cadherin interaction nor the plasma membrane localization of MLK3 is dependent on β-catenin. Collectively, these results indicate a novel function for MLK3 in regulating E-cadherin protein stability, cell adhesion, and ovarian cancer spheroid formation.

## 1. Introduction

Epithelial ovarian cancer is a leading cause of mortality from gynecological cancers worldwide. Ovarian cancer arises from cells that line the ovary as well as from cells originating in the fallopian tube epithelium. Cells from primary tumors can detach and exist as multicellular aggregates or spheroids in ascites fluid of the abdominal cavity. The dissemination of ovarian cancer involves the adhesion of invasive cells of ovarian spheroids to the peritoneal lining, invasion through the mesothelial cell layer, and metastasis to abdominal and pelvic organs [1–3]. Histological types of ovarian cancer are high-grade serous, low-grade serous, clear cell, endometrioid and mucinous carcinomas. High-grade serous ovarian cancers are the most common subtype; they are characterized by rapid progression, frequent TP53 mutations, and high sensitivity to chemotherapy [4]. In contrast, low-grade serous ovarian cancers are rare, develop slowly, have frequent mutations in KRAS and BRAF, rarely have TP53 mutations, and are more resistant to standard chemotherapy [4–6]. Endometrioid, clear cell and mucinous ovarian carcinomas are slow-growing, and often exhibit chemo-resistance in advanced stages [7–9]. Mutations in TP53, PI3KCA, and KRAS are also present in these tumors at varying frequencies depending on the tumor type [7–9].

The serine/threonine mammalian mitogen-activated protein kinase (MAPK), mixed lineage kinase 3 (MLK3), is a MAPK kinase kinase (MAP3K) that regulates migration, invasion and/or proliferation in multiple cancers inclu ovarian, breast, liver, colon and gastric cancer [10–18]. As a MAP3K, MLK3 phosphorylates and activates MAPK kinases (MAP2Ks) MKK4/7 and MKK3/6, leading to the activation of the mammalian stress-signaling MAPK pathways, c-Jun N-terminal kinase (JNK) and p38, respectively [19–22]. In addition, MLK3 can positively regulate the mammalian extracellular signal-regulated kinase (ERK) MAPK pathway through activation of the MAP2K, MEK, or through a kinase-independent regulation of the B-Raf MAP3K in human cancer cell lines [23,24].

MLK3 protein was found to be more highly expressed in ovarian cancer cells in comparison to immortalized ovarian epithelial cells [11]. Furthermore, MLK3 siRNA knockdown in ovarian cancer SKOV3 cells inhibits cell invasion and proliferation [11,24]. Notably, MLK3 expression is also required for matrix metalloproteinase (MMP) −1, −2, −9, and −12 expression and MMP-2 and MMP-9 activities, which are critical mediators of ovarian cancer cell invasion [11,25].

E-cadherin is a calcium-dependent, cell adhesion transmembrane protein that functions in formation and maintenance of adherens junctions in epithelial cell layers [26]. The adherens junctions are cell-cell adhesion complexes comprised of E-cadherin transmembrane proteins that dimerize through their extracellular domains with E-cadherin on adjacent cells. This interaction is specifically mediated by the five extracellular cadherin repeat domains of E-cadherin [27]. Furthermore, the intracellular domains of E-cadherin bind to p120-catenin, β-catenin and α-catenin [28–31]. β-catenin binding stabilizes E-cadherin at the plasma membrane, and the E-cadherin/β-catenin complex is crucial in maintaining stable cell-cell adhesions [28]. Phosphorylation of the E-cadherin cytoplasmic tail by CKII increases E-cadherin binding to β-catenin and promotes strong cell-cell adhesions [32,33].

The loss of E-cadherin expression and dysregulation of adherens junctions is strongly correlated with enhanced migration and cancer cell invasion [34–36]. However, contrary to its function as a cell adhesion protein that limits invasion, recent studies indicate a requirement for E-cadherin in ovarian cancer cell migration, invasion, and proliferation [37,38].

It has been demonstrated that MLK3 regulates β-catenin expression and stabilization in different types of cancer cells, including prostate and breast cancers [39]. MLK3 can interact with and phosphorylate β-catenin, and MLK3 kinase activity is required for stabilizing β-catenin protein. Moreover, MLK3 and β-catenin have similar localization at the plasma membrane and membrane ruffles in HeLa cells [39]. MLK3 is required for ovarian cancer cell invasion and proliferation; however, its role in cell adhesion is unclear [11,24,40]. We investigated the function of MLK3 in regulating E-cadherin and ovarian cancer cell-cell adhesion. The formation of ovarian spheroids, which is mediated by cell-cell adhesions, was dramatically inhibited in ovarian cancer SKOV3, TOV112D, and OVCAR3 cells with MLK3 siRNA knockdown or treated with the MLK3 kinase inhibitor CEP1347; and spheroid formation was restored with E-cadherin overexpression. Ovarian cancer cells with MLK3 knockdown had reduced E-cadherin protein and accelerated E-cadherin protein turnover. Additionally, an interaction between MLK3 and E-cadherin, independent of β-catenin, was observed by co-immunoprecipitation from cell lysates and by *in vitro* binding assays using purified recombinant proteins. Moreover, MLK3 phosphorylated E-cadherin in an *in vitro* kinase assay. MLK3 is localized in the cytoplasm and nucleus, and like E-cadherin, is localized to the plasma membrane at regions of cell-cell contact. Together, these results reveal a novel role for MLK3 in the regulation of E-cadherin stability, mediating cell adhesion and promoting ovarian cancer spheroid formation, which are integral to the development and dissemination of ovarian cancer.

## 2. Materials and methods

### 2.1. Cell culture

Human ovarian cancer TOV112D, SKOV3 and OVCAR3 cells were from the American Type Culture Collection (Manassas, VA, USA). SKOV3 and TOV112D cells were cultured in Dulbecco’s modified Eagle’s medium (Hyclone, Logan, UT, USA) supplemented with 10 % calf serum, l-glutamine, and penicillin/streptomycin. OVCAR3 cells were cultured in Roswell Park Memorial Institute (RPMI) 1640 (Hyclone, Logan, UT, USA) medium supplemented with 20 % calf serum, insulin, l-glutamine, and penicillin/streptomycin. Cells were cultured at 37 °C in a humidified incubator with 5 % CO_2_.

### 2.2. siRNA and plasmid DNA transfections

Nonspecific (NS) and MLK3 siRNA oligos 1 and 2 were from Dharmacon (Lafayette, CO, USA) and target sequences are as previously described [40]. β-catenin siRNA oligo 1 (6225) and 2 (6238) were from Cell Signaling Technology (Danvers, MA, USA). Cells were transfected with 150 nM MLK3 siRNA for 24 h, or with 150 nM β-catenin siRNA for 72 h using Lipofectamine 2000 (Invitrogen-Thermo Fisher Scientific, Waltham, MA, USA) according to the manufacturer’s instructions. Mammalian expression plasmid pCMV-MYC-E-cadherin (Origene Technologies Inc., Rockville, MD, USA) was used for the transient expression of human MYC-E-cadherin in ovarian cancer cells with Lipofectamine 2000.

### 2.3. Immunoblotting

Whole cell extracts were prepared and separated by SDS-PAGE. Proteins were transferred to a polyvinylidene difluoride (PVDF) membrane (MilliporeSigma, Burlington, MA, USA) and blocked with 5 % nonfat dry milk in phosphate-buffered saline (PBS). Immunoblotting was performed with the following primary antibodies from Santa Cruz Biotechnology (Dallas, TX, USA): MLK3 (H-3), and β-catenin (12F7). Other antibodies used for immunoblotting were β-Actin (2D4H5) and MYC (1A5A2) (Proteintech Group, Rosemont, IL, USA); E-cadherin (610182; BD Biosciences, Franklin Lakes, NJ, USA), phospho-Ser/Thr (PM3801; ECM Biosciences), FLAG (200474; Agilent Technologies, Santa Clara, CA, USA), phospho-c-Jun Ser63 (9261) and GST (2625) (Cell Signaling Technology, Danvers, MA, USA). The membranes were incubated with one of the following horseradish peroxidase-conjugated secondary antibodies: Immun-Star goat anti-mouse, Immun-Star goat anti-rabbit (Bio-Rad, Hercules, CA, USA), or goat anti-rat (Thermo Fisher Scientific, Waltham, MA, USA). Next, the membranes were incubated with Immobilon chemiluminescence detection solutions (MilliporeSigma, Burlington, MA, USA) and exposed to X-ray film.

### 2.4. Immunofluorescence

Cells were cultured on sterile coverslips, followed by fixation and permeabilization with 100 % acetone for 5 min at −20 °C. The cells were blocked with 10 % goat serum in PBS for 30 min at room temperature. The coverslips were then incubated with primary antibodies (in PBS with 5 % goat serum) at 37 °C for 1 h using the following dilutions: 1:140 β-catenin (71–2700; rabbit) and 1:125 E-cadherin conjugated with e-Fluor 660 (50-3249-82; rat) from Invitrogen, Thermo Fisher Scientific; and 1:75 MLK3 (H3; mouse, Santa Cruz Biotechnology, Dallas, TX, USA). Slides were washed 3 times for 5 min each. The secondary antibodies, anti-rabbit IgG-Alexa Fluor 568 and anti-mouse IgG-Alexa Fluor 488 (Life Technologies, Carlsbad, CA, USA), were used at a 1:200 dilution in 3 % goat serum in PBS, and the slides were incubated in the dark at room temperature for 1 h, followed by 3 washes in PBS for 10 min each. Coverslips were mounted with Fluoromount G with DAPI mounting medium (00-4959-52; Thermo Fisher Scientific) to preserve the samples and stain the nuclei. Images were captured using a Leica TCS SP5 confocal microscope. Scale bars are indicated in each image, and intensity of fluorescence was measured using ImageJ (NIH).

### 2.5. Immunoprecipitation and in vitro binding assay

Immunoprecipitation was performed from SKOV3, TOV112D, and OVCAR3 cells transfected with nonspecific (NS) or β-catenin oligo 1 or 2 for 72 h. Cells were lysed in IP lysis buffer (20 mM Tris-HCl pH 7.4, 2 mM EGTA, 10 mM MgCl_2_, 0.1 % β-Mercaptoethanol (βMe), 1 % Triton X-100, 100 μM PMSF, 1 μM aprotinin, 2 μM leupeptin and 2 μM pepstatin) on ice. The lysates were centrifuged, and the supernatants were incubated with the respective antibodies (diluted 1:200 in lysis buffer) and A/G PLUS agarose beads (Santa Cruz Biotechnologies, CA, USA) with rotation at 4 °C for 2 h. The beads were washed with high-salt wash buffer (same as lysis buffer but with 0.1 % Triton-X-100 and 0.5 M LiCl) and then wash buffer (same as lysis buffer but with 0.1 % Triton-X-100 and no LiCl). The beads were then suspended in 6× SDS sample buffer, boiled for 5 min and subjected to immunoblotting.

For binding assays, purified, recombinant human GST-MLK3 protein (0.2 μg) (Life Technologies, Carlsbad, CA) was incubated with purified, recombinant human FLAG-E-cadherin protein (0.2 μg) (Origene Technologies Inc., Rockville, MD, USA) in cell lysis buffer as described for immunoprecipitations. FLAG-E-cadherin was immunoprecipitated and the immunoprecipitates were immunoblotted with GST antibody to detect GST-MLK3, and FLAG antibody to detect the immunoprecipitated FLAG-E-cadherin. Input samples were also immunoblotted with GST and FLAG antibodies.

### 2.6. Cell treatments

For cycloheximide (CHX) experiments, cells were transfected with nonspecific (NS) or MLK3 siRNA oligo (1 or 2) and treated with 50 μM CHX for 0, 6, 12 and 24 h time periods. Cell extracts were prepared at the end of each time-period for analysis by immunoblotting. For treatment with the MLK3 inhibitor CEP1347, cells were treated with 10 μM CEP1347 for 24 h and then cell extracts were prepared. CEP1347 was also included in the hanging drop media for spheroid formation assays.

### 2.7. In vitro kinase assay

Immunopurified wildtype human FLAG-MLK3 (FLAG-MLK3-WT) or kinase-dead (K144R) FLAG-MLK3 (FLAG-MLK3-KD) were incubated with 0.3 μg purified, recombinant, human FLAG-E-cadherin (Origene Technologies Inc., Rockville, MD, USA) that was suspended in kinase assay buffer (20 mM MOPS, pH 7.2, 2 mM EGTA, pH 8.0, 10 mM MgCl_2_, 0.1 mM DTT, and 0.1 % Triton X-100) with 100 μM ATP and 10 mM MgCl_2_. Kinase assays were performed for 30 min at 30 °C and the reactions were stopped with 1× SDS sample buffer and heated at 95 °C for 5 min. The samples were subjected to SDS-PAGE and immunoblotting with phospho-serine/threonine antibody (PM3801; ECM Biosciences, Davis, CA, USA) to detect phosphorylated E-cadherin.

### 2.8. Real-time quantitative polymerase chain reaction (qRT-PCR)

Total RNA was isolated from cells using TRIzol reagent (Thermo Fisher Scientific). RNA was reverse-transcribed into cDNA using the Luna Script Reverse Transcription System according to the manufacturer’s instructions (New England Biolabs, Ipswich, MA, USA). qRT-PCR was carried out using SYBR Green-based detection and the following primer sequences (human) were used: MLK3 forward 5′-GTCATGGAATGGCAGTGG-3′ and reverse 5′-CACGGTCACCCTTCCTCA-3′; E-cadherin forward 5’-GAGAGCTACACGTTCACGGT-3′ and reverse 5′-AATTTCTCGGCCCCTTTCCAA-3′; and GAPDH forward *5’*-CTAGGCGCTCACTGTTCTCTC-*3*′ and reverse *5’*-GTCCGAGCGCTGACCTT-3′. Relative gene expression levels were calculated using GAPDH as the housekeeping gene for internal reference.

### 2.9. Spheroid formation assay

SKOV3, TOV112D and OVCAR3 cells were transfected with NS or MLK3 siRNA oligos with or without MYC-E-cadherin plasmid, or treated with 10 μM CEP1347 (Tocris Bioscience/Bio-Techne, Minneapolis, MN, USA) for 24 h. Spheroids were prepared using the hanging drop method as described previously [41]. CEP1347 was included in the media of the hanging drop for spheroid formation. The spheroids were counted, and images were captured with a light microscope at 40× magnification.

### 2.10. Quantification and statistical analysis

Immunoblots are representative of those from three independent experiments and quantification and statistical analyses were performed on three independent biological replicates. Densitometric analysis of immunoblots was performed using ImageJ software from the National Institutes of Health (NIH). The protein signal was normalized to β-Actin and then compared to the appropriate control. For all scatter plot bar graphs generated using GraphPad Prism software (version 10.5.0; GraphPad Software, Inc), bars represent mean ± SD. Statistical significance between two conditions was assessed using an unpaired, two-tailed Student’s *t*-test, *p*-value less than 0.05 is considered significant.

## 3. Results

### 3.1. MLK3 is required for ovarian cancer spheroid formation

**Fig. 1.**
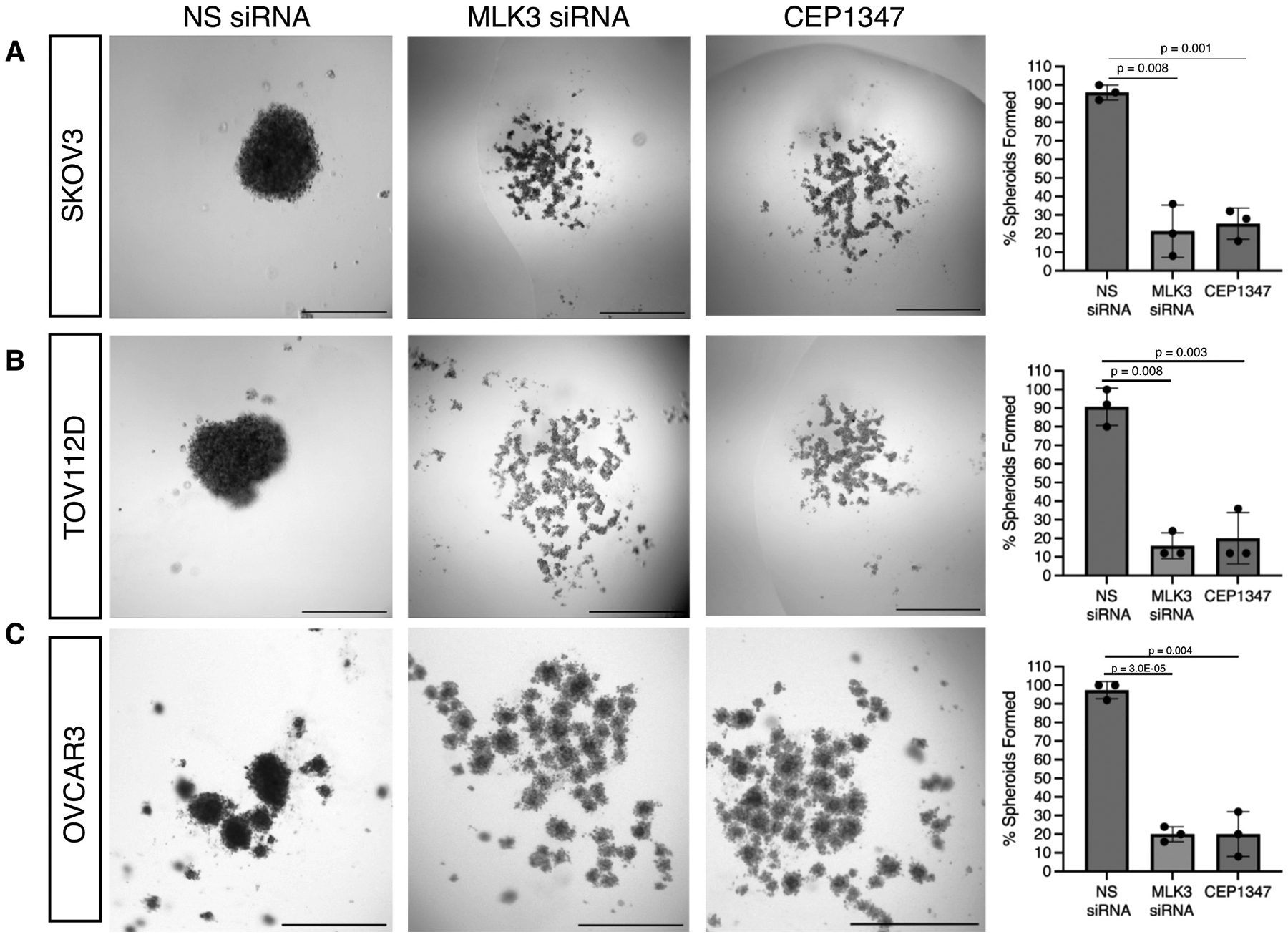
Ovarian cancer spheroid formation is suppressed by MLK3 knockdown or kinase inhibition. (A-C) OVCAR3, SKOV3, and TOV112D cells transfected with NS siRNA, MLK3 siRNA, or treated with 10 μm MLK3 kinase inhibitor CEP1347. Spheroids were formed in hanging drops (left). Data represent mean ± SD of the percentage of spheroids formed (*n* = 3); Student’s *t*-test *P* value is indicated (right). Scale bars represent 100 μm.

The formation of ovarian cancer spheroids involves stable cell-cell adhesions that are mediated by E-cadherin [37,42]. We previously demonstrated that MLK3 siRNA knockdown or MEK kinase inhibition disrupts spheroid formation in TOV112D and SKOV3 ovarian cancer cells [41]. However, the mechanism by which MLK3 regulates spheroid formation is unclear. Here we investigated the effect of MLK3 siRNA knockdown or kinase inhibition on OVCAR3, TOV112D and SKOV3 ovarian cancer spheroid formation. These cell lines were selected to evaluate the impact of MLK3 inhibition on spheroid formation across distinct ovarian cancer subtypes. We chose the high-grade serous ovarian cancer cell line OVCAR3 (HGSOC; TP53 mutation), and the non-serous, advanced stage, ovarian cancer cell lines SKOV3 (clear cell; TP53 null) and TOV112D (endometrioid; TP53 missense mutation). SKOV3, TOV112D and OVCAR3 cells were transfected with siRNA oligos (nonspecific (NS) or MLK3) or treated with MLK3 kinase inhibitor CEP1347 for 24 h. The cells were then plated in hanging drops for spheroid formation (48 h). Both MLK3 knockdown and MLK3 kinase inhibition significantly reduced OVCAR3, TOV112D and SKOV3 spheroid formation indicating that MLK3 kinase activity is required for cell-cell adhesion and formation of ovarian cancer spheroids (Fig. 1).

### 3.2. MLK3 regulates E-cadherin protein levels in ovarian cancer cells

**Fig. 2.**
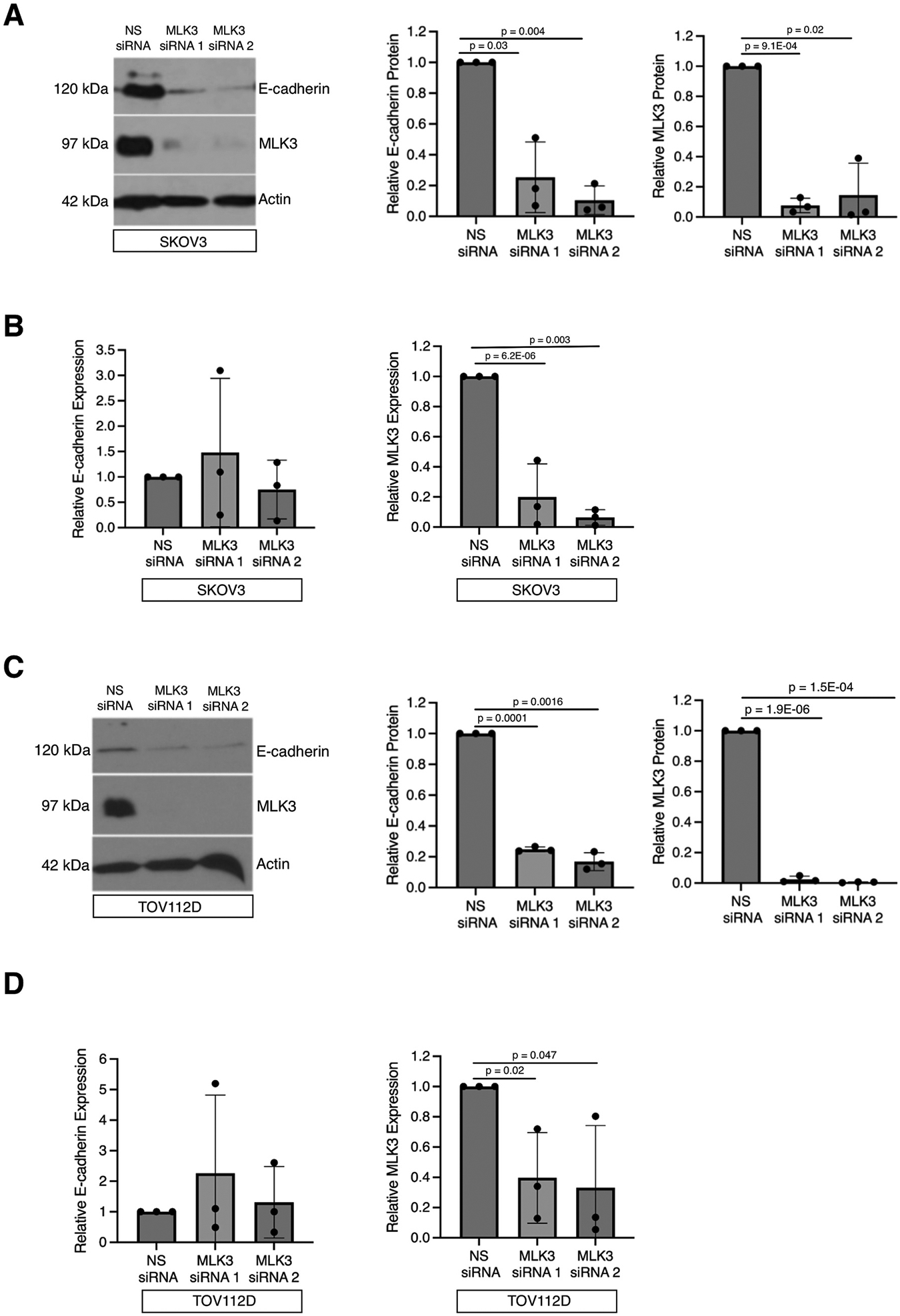
MLK3 knockdown reduces E-cadherin protein levels without altering E-cadherin gene expression. (A, C) Western blot analysis of E-cadherin, MLK3 and β-Actin in SKOV3 and TOV112D cells that were transfected with NS or MLK3 siRNA. (B, D) qRT-PCR analysis of E-cadherin and MLK3 gene expression from SKOV3 and TOV112D cells that were treated as in A and C. Data represent mean ± SD (n = 3); Student’s t-test *P* value is indicated.

E-cadherin is integral in mediating epithelial cell adhesion; and the E-cadherin-β-catenin interaction is critical for stabilizing both proteins in adherens junctions [33]. Furthermore, MLK3 can phosphorylate β-catenin *in vitro* and increase the stability of β-catenin protein [39]. To gain an understanding of how MLK3 regulates cell-cell adhesion, we investigated whether MLK3 modulates E-cadherin protein levels in ovarian cancer cells. SKOV3 and TOV112D cells were transfected with either NS or MLK3 siRNA (oligo 1 or 2) for 24 h, and E-cadherin protein levels were analyzed by immunoblotting. SKOV3 and TOV112D cells with MLK3 siRNA knockdown had significantly reduced E-cadherin protein in comparison to control cells transfected with NS siRNA (Fig. 2A and C). Notably, MLK3 siRNA knockdown in SKOV3 or TOV112D did not significantly affect E-cadherin gene expression as measured by qRT-PCR (Fig. 2B and D). These findings point to a positive regulatory role for MLK3 in maintenance of E-cadherin protein levels in ovarian cancer cells.

### 3.3. MLK3 stabilizes E-cadherin protein in ovarian cancer cells

**Fig. 3.**
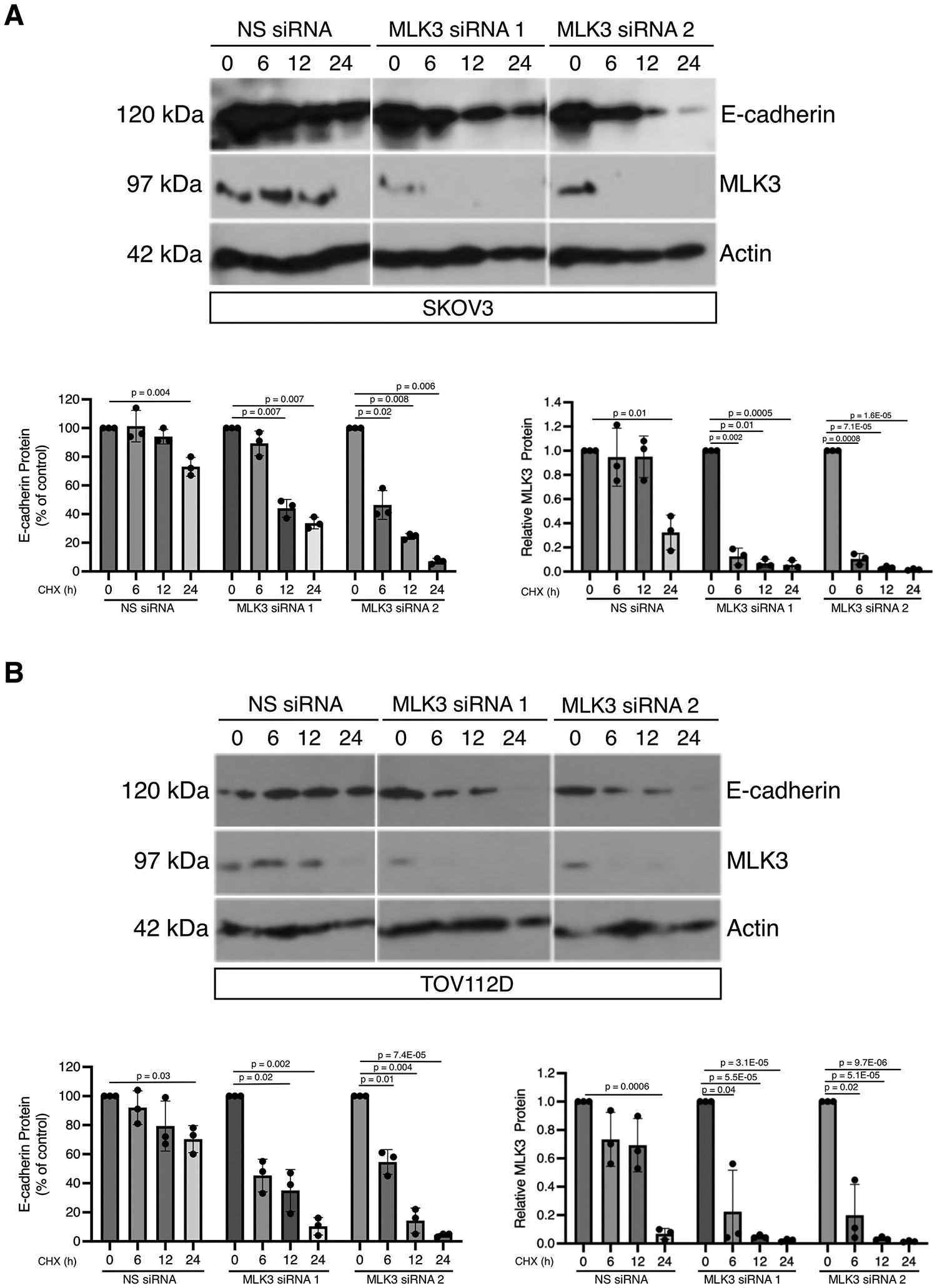
MLK3 knockdown accelerates E-cadherin protein turnover in ovarian cancer cells. (A, B) Western blot analysis of E-cadherin, MLK3 and β-Actin in SKOV3 and TOV112D cells transfected with NS or MLK3 siRNA and treated with cycloheximide (CHX) for different time periods as indicated. Data represent mean ± SD (n = 3); Student’s t-test *P* value is indicated.

Given the effect of MLK3 on E-cadherin protein levels, we sought to determine whether MLK3 affects E-cadherin protein stability. Cycloheximide (CHX) time-course experiments were performed to analyze the turnover of E-cadherin protein in cells with or without MLK3 siRNA knockdown. CHX inhibits protein synthesis by blocking protein translation, and it is used to analyze protein degradation over time and protein half-life [43]. SKOV3 and TOV112D cells were transfected with either NS or MLK3 siRNA (oligo 1 or 2), and treated with CHX for 0, 6, 12 and 24 h. Endogenous E-cadherin and MLK3 proteins in cell lysates were detected by immunoblotting. E-cadherin protein levels gradually declined over the 24 h CHX time course in both MLK3 and NS siRNA-transfected cells. Notably, in SKOV3 cells with MLK3 siRNA knockdown, a rapid decline of E-cadherin protein was observed, with less than 33 % of the protein remaining after 24 h of CHX treatment (siRNA 1 and 2). In contrast, cells transfected with NS siRNA had approximately 73 % of E-cadherin protein remaining after 24 h of CHX treatment (Fig. 3A). Similar results were observed with TOV112D cells (Fig. 3B). These findings indicate that MLK3 expression is required for *E*-cadherin protein stability.

### 3.4. E-cadherin and MLK3 are associated in ovarian cancer cells

**Fig. 4.**
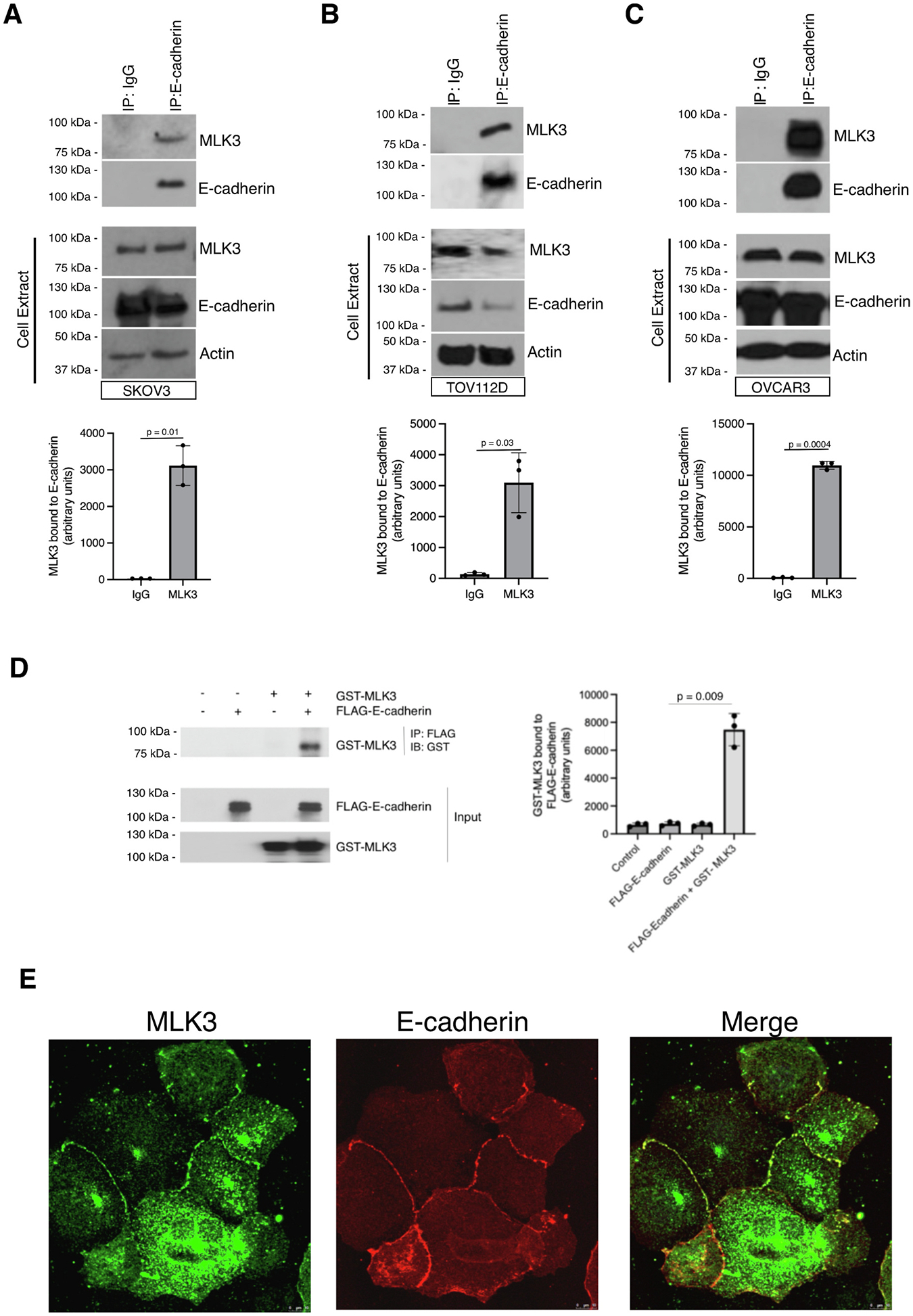
MLK3 interacts with E-cadherin in ovarian cancer cells. (A-C) Western blot analysis of endogenous E-cadherin and MLK3 co-immunoprecipitates from SKOV3, TOV112D, and OVCAR3 cells lysates. D) Purified recombinant FLAG-E-cadherin and GST-MLK3 proteins were combined in an *in vitro* binding assay. Western blot analysis of GST-MLK3 and FLAG-E-cadherin from FLAG-E-cadherin immunoprecipitates and input samples. (E) Immunofluorescence staining of E-cadherin (red) and MLK3 (green) and DNA (DAPI; grey) in OVCAR3 cells. The scale bars are indicated in the figure. Data represent mean ± SD (n = 3); Student’s t-test *P* value is indicated.

Because our results indicate that MLK3 regulates E-cadherin protein levels, we investigated whether MLK3 is associated with E-cadherin in ovarian cancer cells. Co-immunoprecipitations of E-cadherin and MLK3 were performed with TOV112D, OVCAR3 and SKOV3 cell lysates. Endogenous E-cadherin was co-immunoprecipitated with endogenous MLK3 in all three ovarian cancer cell lines, which indicates that MLK3 is associated with E-cadherin in ovarian cancer cells (Figs. 4A–C). Furthermore, in an *in vitro* binding assay, recombinant, purified GST-MLK3 and FLAG-E-cadherin proteins were co-immunoprecipitated, which shows that E-cadherin and MLK3 proteins directly interact (Fig. 4D). Consistent with these findings, both endogenous MLK3 (green) and E-cadherin (red) were localized to the plasma membrane and cell-cell junctions in OVCAR3 cells. MLK3 also had cytoplasmic and nuclear localization (Fig. 4E).

### 3.5. β-Catenin is not required for MLK3 binding to E-cadherin

**Fig. 5.**
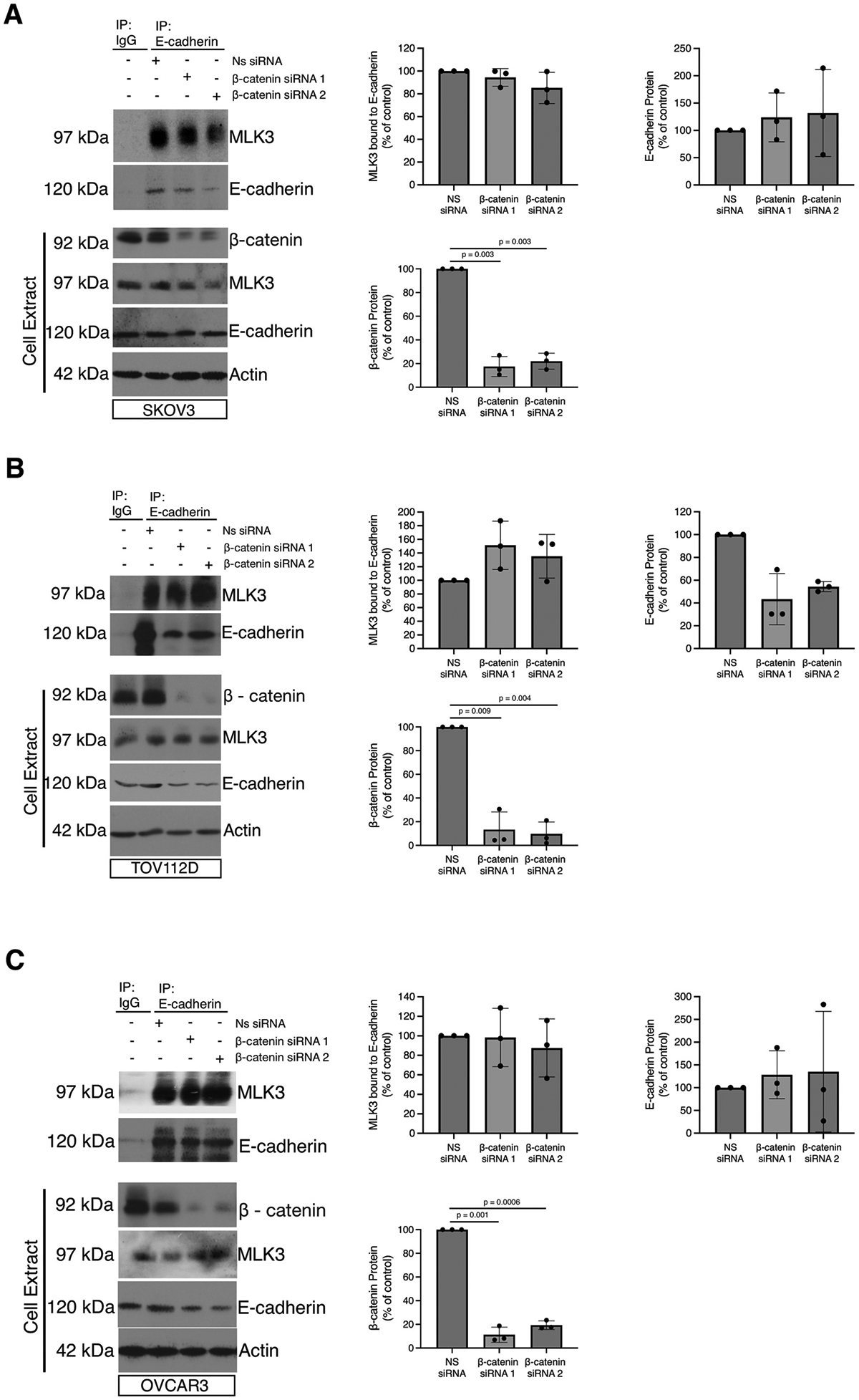
β-catenin is not required for the interaction between MLK3 and E-cadherin. (A-C) Western blot analysis of endogenous E-cadherin and MLK3 in E-cadherin immunoprecipitates from SKOV3, TOV112D, and OVCAR3 cells that were transfected with NS or β-catenin siRNA. Western blotting of β-catenin, MLK3, E-cadherin and β-Actin was performed for whole cell extracts. Data represent mean ± SD (n = 3); Student’s t-test *P* value is indicated.

**Fig. 6.**
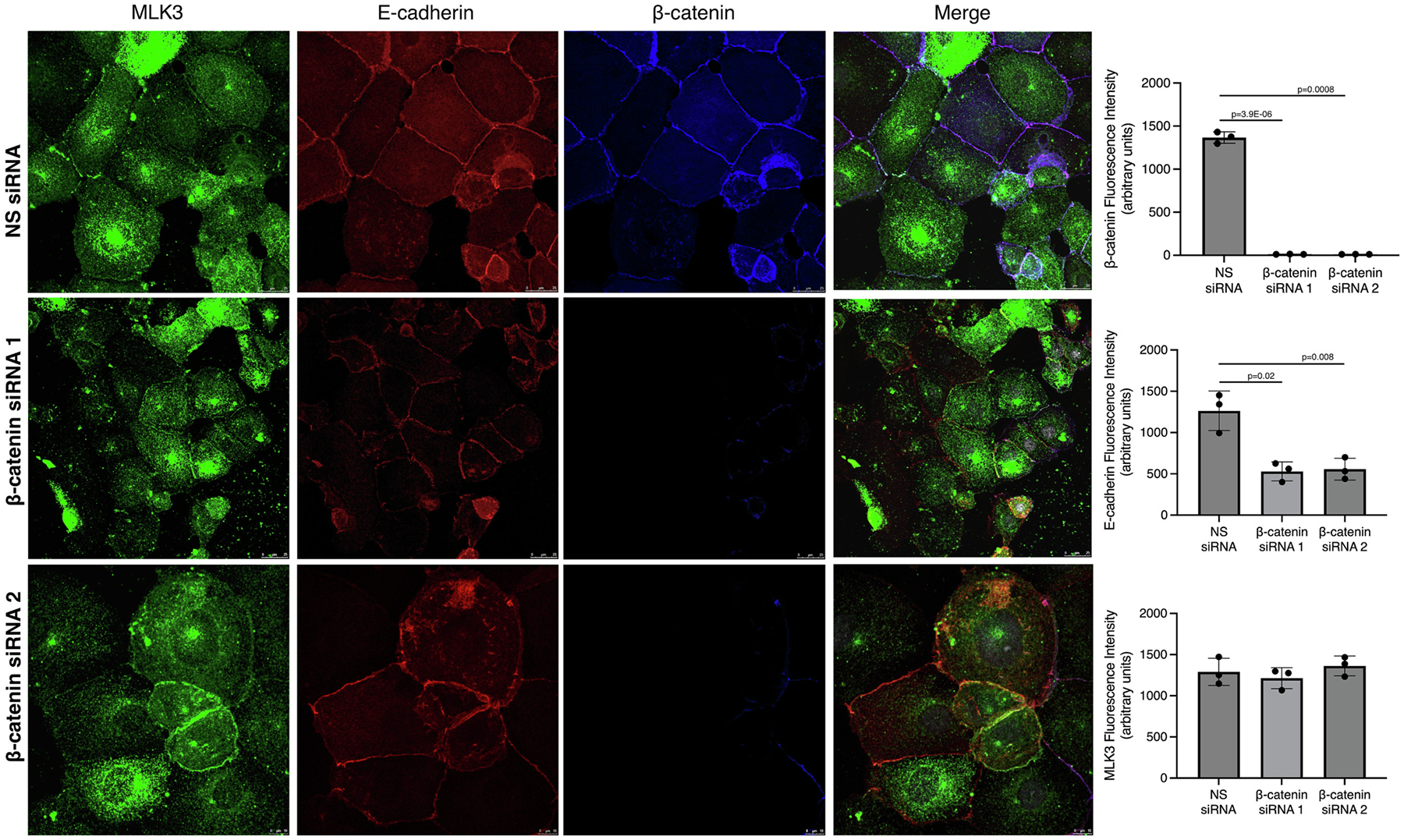
MLK3 localization at cell-cell junctions is not altered by the loss of β-catenin. Immunofluorescent staining of MLK3 (green), β-catenin (blue), E-cadherin (red) and DNA (DAPI, grey) in OVCAR3 cells transfected with NS or β-catenin siRNA. Mean fluorescence intensity of MLK3, β-catenin and E-cadherin was quantified. Data represent mean ± SD (n = 3); Student’s t-test *P* value is indicated. The scale bars are indicated in the figure.

Both E-cadherin and MLK3 are known to interact with β-catenin, which led us to investigate whether β-catenin is required for the interaction between MLK3 and E-cadherin in ovarian cancer cells. Co-immunoprecipitation of endogenous E-cadherin and MLK3 was performed in SKOV3, TOV112D and OVCAR3 cells following β-catenin siRNA knockdown. The loss of β-catenin did not significantly affect the association between MLK3 and E-cadherin in any of the cell lines, indicating that β-catenin is not required for their interaction (Figs. 5A, B and C). Furthermore, to address whether β-catenin is necessary for the localization of MLK3 to cell junctions, immunofluorescent staining of MLK3 and E-cadherin was performed in OVCAR3 cells with β-catenin siRNA knockdown. The loss of β-catenin resulted in approximately a 57 % reduction in E-cadherin protein; however, MLK3 localization at plasma membrane regions of cell-cell contact was unaffected (Fig. 6). These results indicate that MLK3 localization to the plasma membrane and cell-cell contacts is not dependent on its binding to E-cadherin, or on the presence of β-catenin (Fig. 6).

### 3.6. MLK3 kinase activity is required for its regulation of E-cadherin

**Fig. 7.**
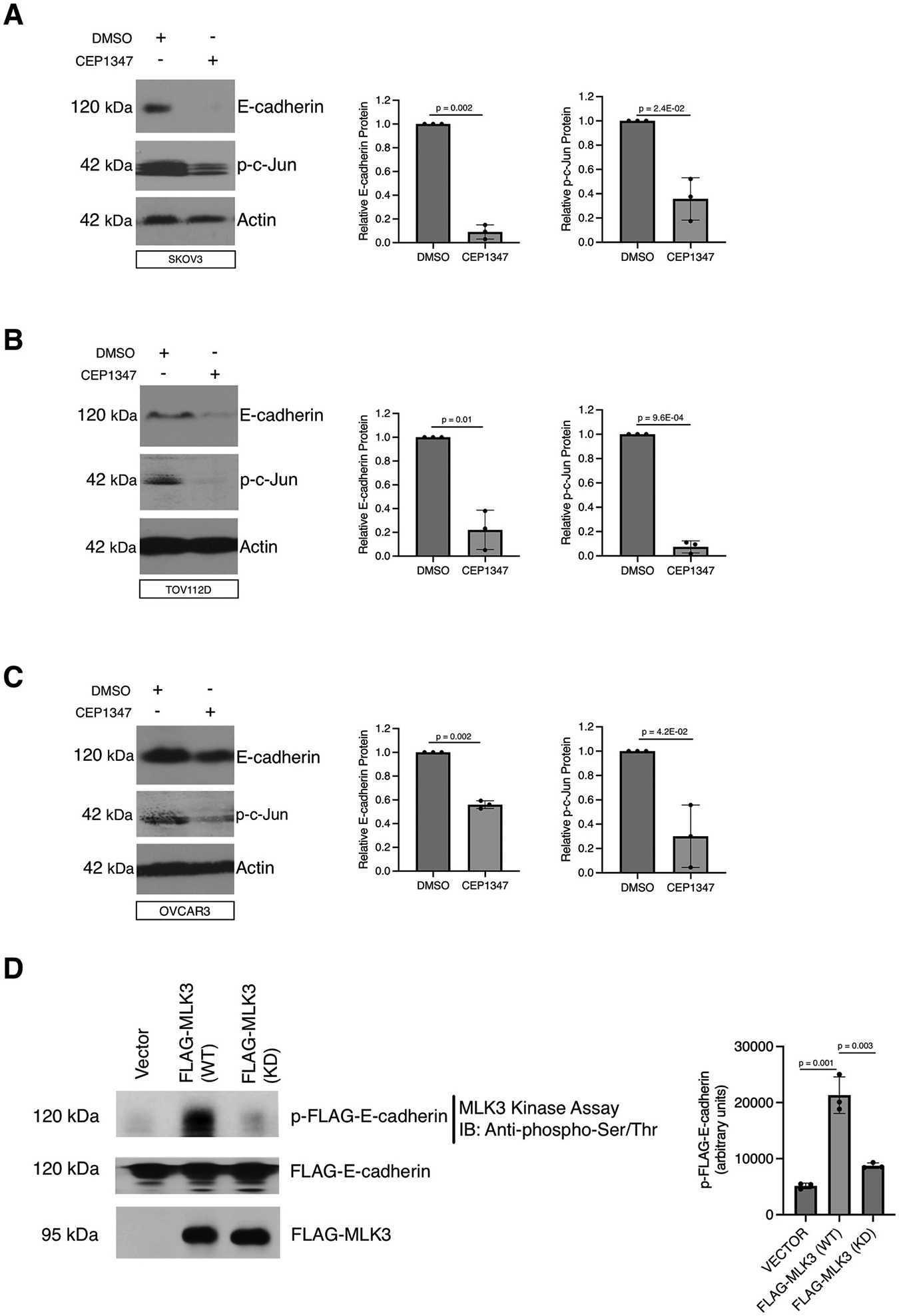
MLK3 kinase activity is required for E-cadherin protein stability. (A-C) Western blot analysis of E-cadherin, p and β-Actin from SKOV3, TOV112D and OVCAR3 cells treated with DMSO or 10 μm CEP1347. (D) *In vitro* kinase assay showing phosphorylation of FLAG-E-cadherin (p-FLAG-E-cadherin) by FLAG-MLK3. Data represent mean ± SD (n = 3); Student’s t-test *P* value is indicated.

To gain a further understanding of how MLK3 regulates E-cadherin protein stability, we assessed the effect of MLK3 kinase inhibition on E-cadherin protein levels. MLK3 kinase activity was inhibited using CEP1347 in SKOV3, TOV112D, and OVCAR3 cells, and E-cadherin protein levels were analyzed by Western blotting. Our results indicate that inhibition of MLK3 kinase activity led to a substantial reduction in E-cadherin protein in all three cell lines (Figs. 7A, B and C). MLK3 kinase activity was efficiently blocked by CEP1347, as measured by immunoblotting of phosphorylated c-Jun which is a downstream target of MLK3/JNK signaling. These results demonstrate that MLK3 kinase activity is important for the regulation of *E*-cadherin protein levels in ovarian cancer cells. Additionally, we examined whether wildtype, active FLAG-MLK3 (FLAG-MLK3 (WT)) could phosphorylate FLAG-E-cadherin directly in an *in vitro* kinase assay. A significant increase in the amount of phosphorylated FLAG-E-cadherin (p-FLAG-E-cadherin) was observed in the samples with active, wildtype FLAG-MLK3 compared to the control or kinase-dead FLAG-MLK3 (FLAG-MLK3 (KD)) (Fig. 7D).

### 3.7. E-cadherin overexpression restores ovarian spheroid formation in cells with MLK3 siRNA knockdown or kinase inhibition

**Fig. 8.**
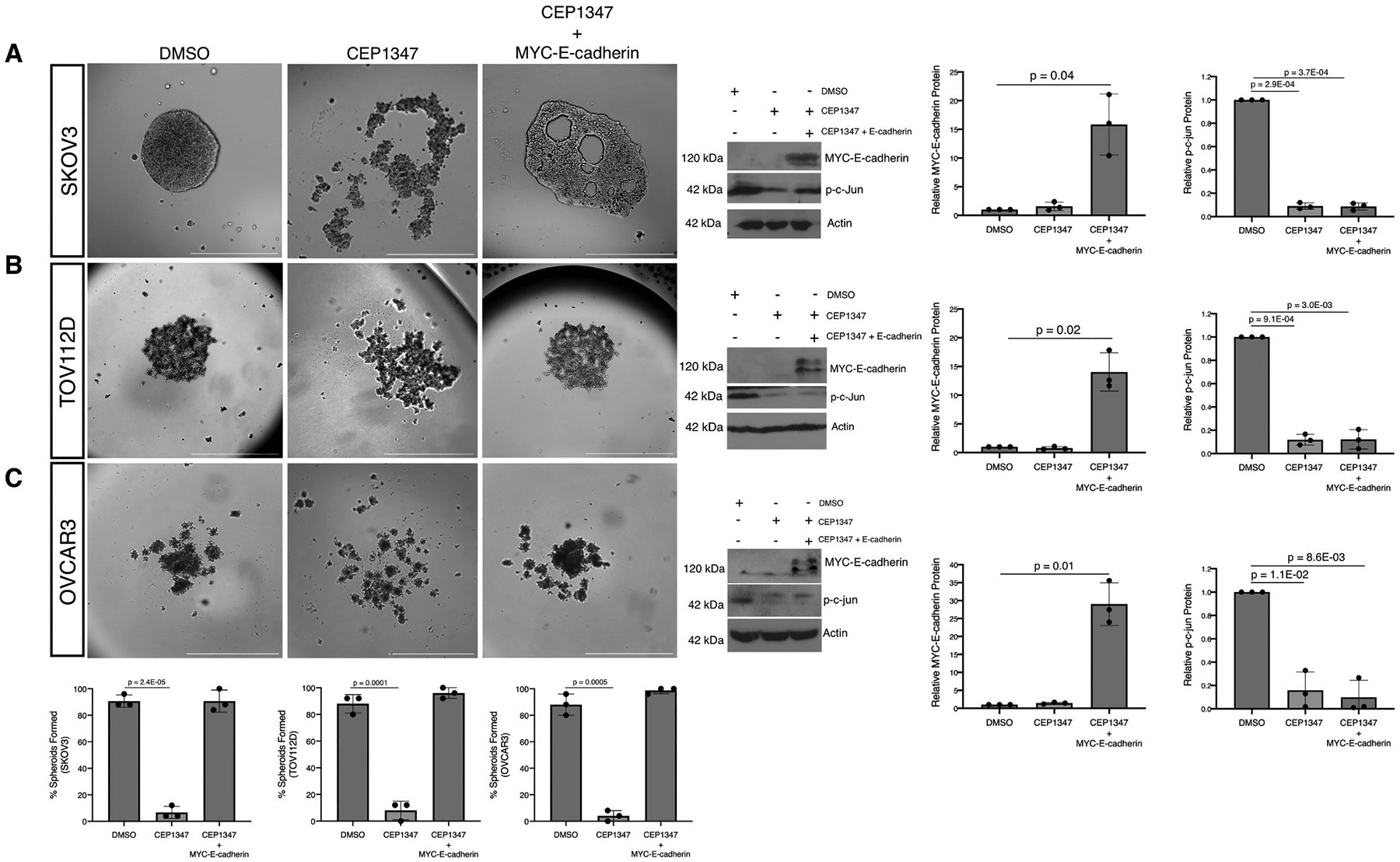
E-cadherin overexpression rescues ovarian spheroid formation suppressed by MLK3 kinase inhibition. (A-C) SKOV3, TOV112D and OVCAR3 cells were treated with DMSO, CEP1347, or CEP1347 and MYC-E-cadherin overexpression for 24 h. Cells were plated in hanging drops and incubated for 48 h to allow spheroid formation (left). Whole-cell extracts were immunoblotted for MYC-E-cadherin, phospho-c-Jun (p-c-Jun), and β-Actin (right). Representative images were captured by light microscopy (40×). Data represent mean ± SD (n = 3); Student’s t-test *P* value is indicated. Scale bars represent 100 μm.

**Fig. 9.**
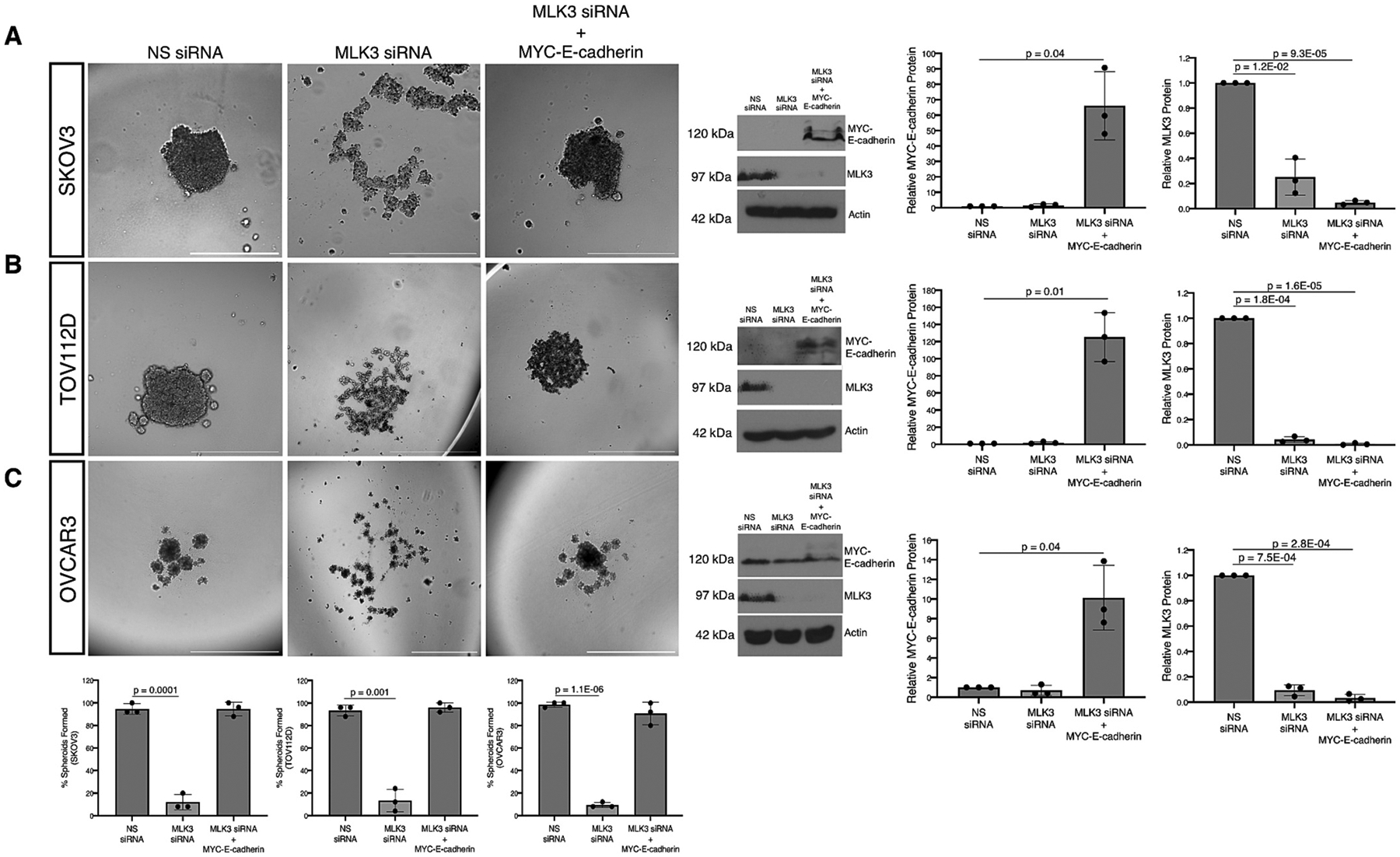
E-cadherin overexpression rescues spheroid formation suppressed by MLK3 knockdown. (A-C) SKOV3, TOV112D and OVCAR3 cells were treated with nonspecific (NS) siRNA, MLK3 siRNA, or MLK3 siRNA and MYC-E-cadherin overexpression for 24 h. Cells were plated in hanging drops and incubated for 48 h to allow spheroid formation (left). Whole-cell extracts were immunoblotted for MYC (E-cadherin), MLK3 and β-Actin (right). Representative images were captured by light microscopy images (40×). Data represent mean ± SD (n = 3); Student’s t-test *P* value is indicated. Scale bars represent 100 μm.

Our results thus far indicate that MLK3 is important for maintenance of E-cadherin protein levels in ovarian cancer cells, and for ovarian spheroid formation. To elucidate whether MLK3-dependent regulation of E-cadherin is necessary for cell-cell adhesion and ovarian spheroid formation, Myc-E-cadherin was overexpressed in SKOV3, TOV112D, and OVCAR3 cells that had MLK3 siRNA knockdown or were treated with CEP1347. Cells with MLK3 kinase inhibition (Fig. 8) or MLK3 knockdown (Fig. 9) had severely impaired spheroid formation with less than 10 % of spheroids formed. Overexpression of Myc-E-cadherin almost fully restored spheroid formation in cells with MLK3 kinase inhibition (Fig. 8) or knockdown (Fig. 9). These results demonstrate that MLK3-dependent regulation of E-cadherin is critical for ovarian cancer cell adhesion and spheroid formation.

## 4. Discussion

We previously observed that both MLK3 knockdown or ERK1/2 pathway inhibition impaired spheroid formation in SKOV3 cells [41]. Our current results indicate that MLK3 knockdown or inhibition of MLK3 kinase activity severely impairs SKOV3, TOV112D and OVCAR3 spheroid formation; and overexpression of E-cadherin in these cells rescues cell adhesion and spheroid formation. The ERK1/2 pathway has also been found to regulate E-cadherin expression and spheroid formation in oral squamous cell carcinoma (SCC) [44]. As MLK3 activates ERK1/2 signaling through both kinase-independent and kinase-dependent mechanisms, we postulate that MLK3-ERK signaling may affect E-cadherin based cell-cell adhesions to support the formation of ovarian cancer spheroids [23,24,45].

In adherens junctions, the cytoplasmic domain of E-cadherin binds to p120-catenin (p120) and β-catenin, which stabilizes E-cadherin at the plasma membrane. In addition, α-catenin binds to β-catenin and associates with the actin cytoskeleton network [31,46,47]. In prostate and breast cancer cells, MLK3 phosphorylates and stabilizes β-catenin [39]. Here, we report that MLK3 kinase activity is necessary for E-cadherin protein stability in three ovarian cancer cell lines. MLK3 can also directly bind to and phosphorylate E-cadherin and has similar localization as E-cadherin in plasma membrane regions of cell-cell contact. Importantly, neither the MLK3-E-cadherin binding nor the plasma membrane localization at cell junctions is mediated by β-catenin. The Rho GTPase, Cdc42, activates MLK3 kinase activity and also facilitates targeting of active MLK3 to the plasma membrane [48]. Additionally, Cdc42 binds to E-cadherin and has critical functions in regulating adherens junctions [49,50]. Therefore, we postulate that recruitment of MLK3 to the plasma membrane and adherens junctions may be mediated by its interaction with Cdc42. Once recruited to adherens junctions, MLK3 may phosphorylate and stabilize E-cadherin. Indeed, phosphorylation of E-cadherin and β-catenin critically regulates their stability, protein-protein interactions and functions in cell adhesion [51–53]. We previously found that MLK3 interacts with the NF2 tumor suppressor protein, Merlin, which is also a component of adherens junction complexes and is required for stable adherens junction formation [45,54,55]. Overall, our findings that MLK3 interacts with E-cadherin and promotes its stability are consistent with MLK3 being a key regulator of E-cadherin-based adherens junction protein complexes.

The interaction between epithelial sheets during Drosophila dorsal closure is dependent upon adherens junction components; and regulated cell adhesion is crucial for epithelial sheet interactions in tissues (44). The MLK ortholog in Drosophila *Slipper* (*slpr*) is required for dorsal closure of the embryo, and *slpr* mutants have a complete failure in dorsal closure [56]. In addition, MLK3 knockout mice have a defect in dorsal closure along the dorsal midline [57]. Our finding that depletion of MLK3 or inhibition of MLK3 kinase activity disrupts ovarian spheroid formation is consistent with these studies and supports a role for MLK3 in cell adhesion in human ovarian epithelial cells [56].

In the dissemination of ovarian cancer, cells from spheroids in ascites fluid invade pelvic and abdominal organs. The attachment of ovarian spheroid cancer cells to the peritoneum involves cadherin-based cell-cell adhesions, and the subsequent invasion and metastasis into tissues requires the activity of MMPs [2,3,58]. Notably, we previously observed that loss of MLK3 blocked MMP activities and expression, and inhibited ovarian cancer cell invasion [11].

In many cancers, the loss of E-cadherin expression leads to dysregulation of adherens junctions and promotes invasion and metastasis [34,59–62]. However, in invasive ductal breast carcinomas, E-cadherin expression promotes metastasis by enhancing survival of metastatic cells [63,64]. Moreover, invasive ovarian tumors often retain some epithelial characteristics including strong expression of E-cadherin [65,66]. In ovarian cancer, a requirement for E-cadherin in cell migration, invasion, and proliferation has been demonstrated [37,38]. Possibly, loss or inhibition of MLK3 in ovarian cancer cells would lead to the downregulation of both E-cadherin and MMP expression and thereby suppress cell invasion. Therefore, targeting MLK3 could be an effective strategy to halt ovarian cancer cell invasion and metastasis as it would disrupt cell-cell adhesions through E-cadherin downregulation and inhibit cell invasion by blocking ERK MAPK signaling and MMP expression.

## 5. Conclusions

Our findings indicate a novel role for MLK3, a MAP3K, in the regulation of the cell adhesion protein E-cadherin, and we propose that MLK3-dependent control of E-cadherin-mediated cell junctions is critical for ovarian cancer spheroid formation. Ovarian spheroids play a key role in the dissemination of ovarian cancer through peritoneal metastasis. Therefore, MLK3 inhibition may serve as a potential therapeutic approach to downregulate E-cadherin, limit spheroid formation and invasion, and ultimately prevent metastasis. Further investigation of the molecular mechanism(s) by which MLK3 regulates E-cadherin and ovarian cancer spheroid formation could facilitate the development of novel therapeutic strategies to treat ovarian cancer.

## Data Availability

Data will be made available on request.

## Abbreviations

MLK3: Mixed Lineage Kinase 3
MAPK: mitogen-activated protein kinase
MAP3K: MAPK kinase kinase
MAP2K: MAPK kinase
JNK c-Jun: N-terminal kinase
siRNA: short interfering RNA
ERK: extracellular signal-regulated kinase
CHX: cycloheximide
